# Effect of food safety training on behavior change of food handlers: A case of orange-fleshed sweetpotato purée processing in Kenya

**DOI:** 10.1016/j.foodcont.2020.107500

**Published:** 2021-01

**Authors:** Derick Nyabera Malavi, George Ooko Abong’, Tawanda Muzhingi

**Affiliations:** a Department of Food Science, Nutrition and Technology, University of Nairobi, P.O Box, 29053-00625, Kangemi, Kenya; b Food and Nutritional Evaluation Laboratory (FANEL), International Potato Centre (CIP), Sub-Saharan Africa (SSA) Regional Office, Old Naivasha Road, P.O Box, 25171-00603, Nairobi, Kenya; c Food Chemistry and Technology Research Centre, Department of Environmental Technology, Food Technology, and Molecular Biotechnology, Ghent University Global Campus, 119-5, Songdomunhwa-Ro, Yeonsu-Gu, Incheon, 21985, South Korea; d Department of Food, Bioprocessing and Nutrition Sciences, North Carolina State University, Raleigh, NC, 27695, USA

**Keywords:** Training, Food safety, Food handler, Hygiene practices, Contamination, Indicator microorganism

## Abstract

Sweetpotato purée processing is new to Kenya and a rapidly growing value addition activity among informal, small, and medium-sized food enterprises (SMEs) in sub-Saharan Africa (SSA). Inadequate knowledge of food safety and poor hygiene practices by food handlers, low level of compliance with Good Manufacturing Practices (GMPs), and microbial contamination are major food safety challenges in orange-fleshed sweetpotato (OFSP) purée processing in Kenya. The extent of food safety training in enhancing food safety in rural-based SMEs and food processing environments has not been fully investigated. This study aimed at evaluating the impact of food safety training on food safety knowledge and hygiene practices of food handlers and in control of microbial contamination in OFSP purée processing in Kenya. Pre- and post-food safety training assessments were conducted to determine food handler's (N = 14) knowledge and practices on food safety. Food, water, and swab samples (n = 62) from the processing environment were collected before and two months after the training and analyzed for food hygiene indicator microorganisms. The findings indicate a significant (*p* < 0.05) improvement in overall food safety knowledge and practices of food handlers after the training. Poor knowledge scores were exhibited on aspects of cross-contamination, cleaning, and sanitation but these significantly (*p* < 0.05) improved after the training. Similarly, microbial counts on food equipment surfaces, installations, personnel hands, and in the final product (OFSP purée) significantly (*p* < 0.05) declined to acceptable levels after the food safety training. Total counts, yeasts and molds, *S*. *aureus,* Enterobacteriaceae, and total coliforms counts in the packaged OFSP purée were 2.6, 1.8, 1.5, 1.9, and 1.2 LOG CFU/g respectively hence suitable for its current application as an ingredient in baked products. The findings from this study indicate food safety training as an appropriate tool for improving food handler's knowledge and hygiene practices as well as enhancing microbial safety and quality of processed foods in SMEs if necessary food safety support resources are provided.

## Highlights

• Orange-fleshed sweet potato purée processing is often faced by food safety challenges.
• Food safety training is proposed to solve these problems.
• Food safety training improves the knowledge and hygiene practices of food handlers.
• Microbial quality and safety of foods improve through food safety training.

## 1 Introduction

Biofortified orange-fleshed sweetpotato (OFSP) is an important food security root crop in sub-Saharan Africa (SSA). It is rich in beta-carotene, a provitamin A carotenoid, and a relatively cost-effective strategy for alleviating Vitamin A deficiency (VAD) in SSA (Low et al., 2017; Kurabachew, 2015; Burri, 2011). OFSP fresh roots are processed into a purée (or mash) used as a wheat flour substitute ingredient in baked and fried products by bakeries in retail chain stores in Kenya (Malavi et al., 2017; Tedesco & Stathers, 2015 February 2015). OFSP purée processing in SSA is currently done by small and medium-sized food enterprises (SMEs) and faces a lot of food safety challenges attributed to low compliance with Good Manufacturing Practices (GMPs), inadequate knowledge on food safety, poor hygiene practices, and contamination from the processing environment (Malavi et al., 2018; Malavi et al., 2017).

It has been well established that failure to comply with food safety regulations in food processing environments often leads to microbial contamination that results in incidences of foodborne diseases (FBD) and food spoilage (Malavi et al., 2018; Mahmoud & Sivakumar, 2014). The World Health Organization (WHO) reports 98% of the global burden of FBD from low and middle-income countries (WHO, 2014). Foods most implicated are highly nutritious vegetables and animal sources of foods. Multiple and frequent foodborne disease outbreaks indicate food safety as a global public health concern. Foodborne diseases cause morbidity, mortality, and reduced socio-economic development (CDC, 2017; WHO, 2015). Several factors such as the use of contaminated raw materials, contaminated equipment, poor personal hygiene, and failure in monitoring critical control points during processing compromise the quality and safety of foods (Mahmoud & Sivakumar, 2014; Roberts et al., 2008). Food safety can, however, be enhanced by creating awareness through training and implementation of stringent hygiene measures along the value chain (production, processing, storage, distribution, and consumption) (Korada et al., 2018).

The roles played by food handlers along the food chain are critical in food safety and quality assurance. Food handlers are known to be carriers of foodborne pathogens hence potential sources of food contamination (Malavi et al., 2018; Soares et al., 2012; Argudin et al., 2010). *Escherichia coli*, *Staphylococcus aureus*, and *Salmonella* spp. are major pathogens associated with violation of food handling practices (Sousa, 2008; Lues & Van Tonder, 2007). Poor personal hygiene and inappropriate food handling practices have been reported as major causes of food contamination by food handlers (Baluka et al., 2015; Gungor & Gokoglu, 2010).

Limited knowledge of food safety and poor hygiene practices among OFSP purée handlers; low compliance with GMPs; and higher levels of microbial contamination in the OFSP purée processing environment have been reported as the major food safety problems in OFSP purée processing in Kenya (Malavi et al., 2018; Malavi et al., 2017; Malavi, 2017). There was an urgent need to improve food safety knowledge and practices of food handlers and control microbial contamination from all food safety risk points in OFSP purée processing to enhance the safety and quality of OFSP purée. Several studies have identified food safety training as an effective tool for improving food handlers' knowledge and practices (Bas et al., 2006, Husain et al., 2016; Soon & Baines, 2012; Kassa et al., 2010; Park et al., 2010; Roberts et al., 2008). It has previously been reported that food safety training can reduce incidences of foodborne illnesses if it increases employee's motivation, attitudes, and the frequency of appropriate food handling practices (Soon et al., 2012; Soon & Baines, 2012). A study by Webb and Morancie (2015), recommend detailed attention on planning, implementation, monitoring, and evaluation of food safety training to improve food safety knowledge among food handlers. Adesokan et al. (2015), also proposes food safety training as an approach for improving food handling practices. On the contrary, another study by Thimoteo et al. (2014) argues that food safety training does not necessarily lead to improved attitudes and practices. These contradicting results indicate the need to further investigate the impact of food safety training on the knowledge and practices of food handlers. There is also a lack of information on the effectiveness of food safety training on controlling microbial quality in food processing. The objective of this study was therefore to evaluate the impact of food safety training on knowledge and practices of food handlers and levels of microbial hygiene indicators in orange-fleshed sweetpotato purée processing in Kenya.

## 2 Materials and methods

### 2.1 Study area and participants

This study was done in an OFSP purée processing plant in Homa Bay County, Kenya. It was the only OFSP purée processing factory operating on a small-scale basis in Kenya. Due to the low number of food-handling personnel at the factory justified by its small-scale operation, exclusive sampling was used to select all the food handlers involved in sweetpotato purée processing. A total of 14 respondents voluntarily agreed to participate in the study. All the respondents were involved in the handling of sweetpotato processing either at washing, peeling, steaming, puréeing, packaging, and storage. The information on the socio-demographic factors including gender, age, education level, work section, previous training in food safety, employment period, and motivation to work in OFSP purée processing was collected for comparison with the food safety knowledge and hygiene practices scores of food handlers at the processing facility.

### 2.2 Training of OFSP purée handlers and assessment of knowledge and hygiene practices

Training modules for the orange-fleshed sweetpotato purée handlers was developed based on literature (Soon & Baines, 2012; Park et al., 2010; Simonne et al., 2010; Seaman & Eves, 2006) and gaps as highlighted in publications by Malavi et al. (2018) and Malavi et al. (2017). A pre-training test with 35 questions on food safety and 13 items on hygiene practices was administered to food handlers to assess their level of food safety knowledge and practices. They were asked to circle the correct answer for each question with choices: True, False and Don't Know for items on food safety knowledge; and Always, Sometimes, Rarely and Never for items on food handling practices. The respondents were then trained on appropriate hygiene practices for enhancing food safety in purée processing. The training covered topics on personal hygiene, cross-contamination, environmental hygiene, food storage, and process control, pest control, cleaning, and sanitation. The training session involved theory classes on food safety and hygiene, pictorial and video demonstrations on personal hygiene, practical demonstrations on handwashing hygiene, cleaning and sanitation of food processing equipment, and environmental hygiene. After the training, a post-test was again administered to food handlers to assess their food safety knowledge only. Several recommendations such as sanitation of equipment, acquisition of more hand washing hygiene resources, water treatment, training of new staff on basic food safety, provision of enough personal protective equipment among others were given to the management team to improve food safety after training.

### 2.3 Post-training food handling practices assessment and environmental hygiene sampling

After the training, follow-ups were done through site visits at the processing plant to observe their hygiene practices. Microbial sampling and reassessment of food handler's practices using a similar questionnaire that was administered during the first phase of the study were done after two months from the last date of the food safety training. It is important to mention that food safety training was immediately conducted (14 days after the first sampling) based on the preliminary results that were later published in studies by Malavi et al. (2018) and Malavi et al. (2017). Therefore, microbial data from the previous study (Malavi et al., 2018) served as the baseline data for the current study. Targeted environmental sampling for assessment of microbial hygiene indicators was used to collect a total of 62 samples based on findings reported by Malavi et al. (2018). Briefly, previously sterilized papers of areas 30 cm^2^ and 60 cm^2^ were used to outline surface areas for swabbing. Buffered swab sponges (3M sponge-sticks) were then used to collect samples from the equipment (30 samples), personnel hands (8 samples), drains (3 samples) walls (3 samples), and floors (3 samples) as described by Malavi et al. (2018) for microbial analysis. Samples from food equipment and installations were collected after cleaning and sanitation while samples from food-handling personnel were collected after handwashing but during working hours. The OFSP samples were collected at four different stages in three different batches: after washing (3 samples), steaming (3 samples), cooling and slicing (3 samples), and packaging (3 samples). Three water samples used for cleaning, processing, and handwashing at the factory were also collected in sterile sample bottles for analysis. All the collected samples were packaged in ice-filled cold boxes and transported to the Department of Food Science, Nutrition and Technology, University of Nairobi, and immediately analyzed for total viable counts (TVC), yeasts, and molds, *Staphylococcus aureus*, Enterobacteriaceae, coliforms and *Escherichia coli.*

### 2.4 Sample preparation and microbiological analysis

Samples were prepared as described in the previous publications (Malavi et al., 2018; Gwida et al., 2014; Gungor & Gokoglu, 2010; Schlegelova et al., 2010; Perez-Diaz et al., 2008). Swab samples from equipment, personnel, floors, and walls were diluted with 90 mL of buffered peptone water (BPW, Oxoid, UK). Twenty-five grams of orange-fleshed sweetpotato samples and 25 mL of water were separately mixed with 225 mL of BPW saline water to make a dilution of 10^−1^. Subsequent serial dilutions were prepared using 0.85% sodium chloride (Oxoid) and analyzed for TVC, yeasts and molds, *S. aureus*, Enterobacteriaceae, total coliforms, and *E. coli* (EC). All the analyses were done in triplicate. Culture plates with counts between 30 and 300 colonies were enumerated and the average was expressed as LOG_10_ colony forming units (LOG_10_ CFU) per g/cm^2^/mL depending on the sample (Malavi et al., 2018).

*Total Viable Counts*: Determination of TVC was done by the spread plate technique on Plate Count Agar (PCA). Exactly 0.1 mL of each diluted sample was pipetted and spread on Plate Count Agar (PCA, LAB, UK) using a sterile disposable spreader. The plates were incubated upside down at 35 °C for 48 h (Malavi et al., 2018; Gungor & Gokoglu, 2010; Liguori et al., 2010; Perez-Diaz et al., 2008).

*Yeasts and Molds*: Analysis of yeasts and molds was also done by the spread plate technique on Potato Dextrose Agar (PDA, Oxoid, Hampshire). The plates were then incubated at 25 °C for 5 days (Malavi et al., 2018; Gungor and Gokoglu (2010).

Enterobacteriaceae*, total coliforms and E. coli*: Enterobacteriaceae family of microorganisms were determined by the spread plate method of samples on Violet Red Bile Glucose (VRBG) Agar (Oxoid) followed by incubation of plates at 37 °C for 24 h as described by Malavi et al. (2018) and Hervert et al. (2016). Total coliforms and *E. coli* were analyzed by plating 0.1 mL of each diluted sample on triplicate Petri dishes with Brilliance *E. coli*/coliform selective agar (Oxoid, Hampshire, England). *Escherichia coli* ATCC 8739 strain (Microbiologics, MN 56303-USA) was used as a positive control. The plates were incubated at 37 °C for 24 h (Malavi et al., 2018; Baluka et al., 2015). Purple colonies were enumerated and recorded as *E. coli* while pink colonies were recorded as other coliforms (Baluka et al., 2015).

*Staphylococcus aureus:* Detection, identification, enumeration and biochemical confirmation of *S. aureus* in the samples was done by spread plate technique on Baird Parker medium followed by incubation at 37 °C for 24 h as described by Mahmoud and Sivakumar (2014); Gungor and Gokoglu (2010). *S. aureus* ATCC 6538 (Microbiologics, MN 56303-USA) was used as a positive control. Confirmation of typical *S. aureus* colonies was done by the coagulase test as described by Malavi et al. (2018).

### 2.5 Statistical analyses

Data analysis was done using Microsoft Excel 365 and the Statistical Package for the Social Sciences (IBM SPSS Statistics for Windows, Version 25.0. Armonk, NY: IBM Corp, 2017) software. Responses for both knowledge and practice questions were coded on a two-point scale with 0 for an incorrect answer and 1 for every correct response. All correct answers were totaled and converted to a percentage score. Frequencies and descriptive statistics were used to summarize scores for each knowledge and food hygiene practice item. The level of knowledge and practices were considered high for scores above 80% and low for scores below 80% based on the previous study (Malavi et al., 2017). The variables in the data sets were tested for normality before conducting further statistical analysis. Non-parametric tests: Mann-Whitney U and Kruskal-Wallis One-way ANOVA tests were used to evaluate whether food safety knowledge and practice mean scores varied with the respondents' socio-demographic characteristics. Wilcoxon Signed Ranks Test was used to compare pre- and post-training food safety knowledge and hygiene practices scores. Parametric tests were used to test statistical significance from the data set on microbial contamination. One-way analysis of variance (ANOVA) and Tukey's test for post hoc analysis was used to test statistical differences and separate mean microbial counts among different samples. Paired samples *t*-test was used to compare mean microbial counts on the equipment, installations, personnel, and in the packaged purée before and after food safety training. The level of statistical significance was set at *p* < 0.05.

## 3 Results and discussion

### 3.1 Influence of socio-demographic factors on food safety knowledge and practices of orange-fleshed sweetpotato purée handlers

A total of 14 food handlers participated in this study. Ten (71.4%) of the respondents were male while four (28.6%) were female; eight (57.1%) of the food handlers were within the age group of 18–25 years and six (42.9%) were above 25 years. Based on the level of education, eight (57.1%) of the food handlers had attained post-primary education. Twelve (85.7%) of the respondents worked in the OFSP washing, peeling, and processing sections. Nine (64.3%) of the food handlers had worked at the purée processing plant for not more than a year. Ten (71.4%) of the food handlers had previously received training in food safety while the other four had not received any training.

Table 1 shows changes in food safety scores as per the socio-demographic factors before and after the food safety training. Female respondents demonstrated good knowledge and better practices than males but it was not significant (*p* > 0.05). Age, level of education, length of employment at the establishment, and previous training in food safety significantly (*p* < 0.05) influenced the knowledge scores of food handlers. Food handlers with less than 6 months of employment at the processing facility had poor knowledge of food safety indicating the value of experience and the need of training new employees. High level of food safety knowledge among OFSP purée handlers with a relatively longer working period at the facility is attributed to their previous exposure to basic food safety training sessions at the plant. Personnel in management demonstrated high levels of food safety knowledge followed by those in processing, peeling, and washing and cooking sections respectively. Motivated food handlers displayed better knowledge and practices than their unmotivated counterparts but it was not significant (*p* > 0.05). Food handling practices in this study were only influenced by the employee's previous training in food safety (*p* = 0.029). Similar to findings by Thomas and Adesina (2015) there was no significant relationship between hygiene practices of food handlers and gender (*p* = 0.945), age (*p* = 0.852), education level (*p* = 0.509) and work experience (*p* = 0.265) in this study.

**Table 1:** Pre-training food safety knowledge and hygiene practices scores of OFSP purée handlers as per the socio-demographic characteristics (N = 14).

Variable		Knowledge			Practices	
		n	Mean ± SD	p-value	Mean ± SD	p-value
Gender	Male	10	77.4 ± 17.0	0.569	73.8 ± 10.4	0.945
	Female	4	85.0 ± 7.5		84.6 ± 14.0	
Age	18–25	8	72.9 ± 15.3	0.043*	70.3 ± 20.5	0.852
	26–35	6	88.6 ± 9.4		83.3 ± 5.3	
Highest Education level	Primary	6	70.0 ± 16.3	0.039**	64.1 ± 42.4	0.509
	Secondary	6	83.8 ± 9.2		78.6 ± 24.0	
	Tertiary	2	95.7 ± 2.0		88.5 ± 16.3	
Job role/work section	Management	2	95.7 ± 2.0	0.166	100.0 ± 0.0	0.218
	Peeling/Washing	5	77.1 ± 18.7		92.6 ± 4.33	
	Cooking	2	68.6 ± 4.0		82.9 ± 20.2	
	Processing	5	80.0 ± 13.4		91.4 ± 4.5	
Have you previous training in food safety?	Yes	10	86.3 ± 10.1	0.015*	89.4 ± 13.0	0.029*
	No	4	62.9 ± 12.1		60.3 ± 32.1	
Employment period	<3 months	4	62.9 ± 12.1	0.031**	69.3 ± 2.7	0.265
	3–6 months	2	65.4 ± 4.04		72.9 ± 10.1	
	7–12 months	3	81.9 ± 14.1		74.7 ± 15.7	
	>1 year	5	86.9 ± 10.0		89.6 ± 2.4	
Are you motivated to work in OFSP processing?	Yes	6	81.9 ± 13.1	0.896	80.1 ± 11.3	0.756
	No	8	77.9 ± 17.00		72.2 ± 5.5	

The values in columns for knowledge and practices indicate mean ± standard deviation.

*/** Mean difference statistically significant (Mann-Whitney U/Kruskal Wallis test at p < 0.05).

### 3.2 Pre- and post-training food safety knowledge responses by orange-fleshed sweetpotato purée handlers

The frequency of responses to food safety knowledge questions before and after training by the OFSP purée handlers is shown in Table 2a, Table 2ba and 2b. Only half of the food handlers knew equipment should always be cleaned and sanitized after use in processing. More than half of them (64.3%) demonstrated poor knowledge of handwashing hygiene practices and 57% did not know proper cooking is effective in destroying most pathogenic and spoilage microorganisms during purée processing. These findings closely relate to findings by Adesokan et al. (2015) and Park et al. (2010) which reported poor knowledge scores by untrained food handlers on temperature control, sources of food contamination, and personal hygiene. An increase in about 90% correct response rate was, however, recorded from all food safety questions after food safety training.

**Table 2a:** Food safety knowledge scores of OFSP purée handlers before and after food safety training (N = 14).

Food Safety Questions	Before Training		After Training	
	Correct Answers (%)	Wrong Answers (%)	Correct Answers (%)	Wrong Answers (%)
1) Microorganisms in food cause both food spoilage and foodborne diseases	92.9	7.1	100.0	0.0
2) Food safety hazards are broadly classified into physical, chemical and biological hazards.	85.7	14.3	100.0	0.0
3) Everyone is responsible for food safety	78.6	21.4	92.9	7.1
4) We should always wash our hands with soap before and after handling sweetpotato roots and purée, after visiting the toilet, after touching or scratching our nose, skin, and mouth and after touching or using our mobile phones	78.6	21.4	85.7	14.3
5) Microbes are on skin, mouth, and nose of healthy sweetpotato purée handlers	78.6	21.4	92.9	7.1
6) Equipment for sweetpotato purée processing should always be cleaned and sanitized after processing	50.0	50.0	78.6	21.4
7) Personal protective clothing/gear includes apron, hairnets and clean closed shoes	85.7	14.3	92.9	7.1
8) To wash hands, I should: Use soap; Scrub my hands and remove dirt from my fingernails for at least 20 s; and rinse under running tap water	64.3	35.7	85.7	14.3
9) Working with rings, necklaces and watches is allowed in purée processing	85.7	14.3	100.0	0.0
10) Processing of sweetpotato purée can be done on dirty equipment	85.7	14.3	100.0	0.0
11) Fresh sweetpotato roots can be stored together with processed purée	92.9	7.1	92.9	7.1
12) I must report to the manager immediately in case I experience signs of a disease such as vomiting and diarrhea	78.6	21.4	92.9	7.1
13) I can smoke, chew or drink while handling sweetpotato roots and the purée	78.6	21.4	85.7	14.3
14) Use of mobile phones is allowed while handling sweetpotato and processed purée	85.7	14.3	92.9	7.1
15) I can handle sweetpotato and processed purée with an open wound from a hand injury, boil or skin infection	85.7	14.3	100.0	0.0
16) I should report to work when I am clean and presentable	85.7	14.3	85.7	14.3
17) Blowing my nose and coughing is allowed while handling sweetpotato and the purée	71.4	28.6	92.9	7.1

**Table 2b:** Food safety knowledge scores of OFSP purée handlers before and after food safety training (N = 14).

Food Safety Questions	Before Training		After Training	
	Correct Answers (%)	Wrong Answers (%)	Correct Answers (%)	Wrong Answers (%)
18) I can wear my lab coat and safety shoes outside the processing area	92.9	7.1	92.9	7.1
19) My fingernails need to be kept short and clean always	71.4	28.6	100.0	0.0
20) Sanitation of equipment for sweetpotato purée processing can be done using hot water	71.4	28.6	100.0	0.0
21) Sanitation can only be done after cleaning the equipment	92.9	7.1	100.0	0.0
22) Sanitation destroys/removes/kills 99.999% of disease-causing microorganisms from equipment surfaces	78.6	21.4	100.0	0.0
23) Waste from spilled sweetpotatoes, purée, and peels should immediately be picked from the floor and disposed of in a labeled waste bin.	78.6	21.4	100.0	0.0
24) Contamination in sweetpotato purée can occur when (a)raw sweetpotato roots are mixed with the purée; (b)equipment for processing are not cleaned and sanitized; (c) when food handlers fail to practice appropriate hygiene practices	71.4	28.6	100.0	0.0
25) Rotten sweetpotato roots should not be separated from ‘good’ roots	78.6	21.4	85.7	14.3
26) It is not important to wash roots before peeling/cooking	71.4	28.6	92.9	7.1
27) The surface of OFSP roots should be scrubbed with a sanitized brush to remove soil	78.6	21.4	78.6	21.4
28) Water for washing roots needs not to be changed regularly	50.0	50.0	85.7	14.3
29) Damaged/bruised areas of OFSP roots should be removed with a knife before cooking or puréeing	71.4	28.6	92.9	7.1
30) It is not necessary to wash knives and roots that fall on the floor during peeling	85.7	14.3	92.9	7.1
31) The soil on sweetpotato roots could be carrying harmful bacteria	71.4	28.6	85.7	14.3
32) Cooking process DOES NOT destroy microorganisms on sweetpotato roots	57.1	42.9	78.6	21.4
33) I cannot contaminate the purée when using the puréeing machine or during packaging if I do not wash my hands properly.	71.4	28.6	78.6	21.4
34) I can eat/taste sweetpotato or purée while in the processing room	85.7	14.3	92.9	7.1
35) It is right coughing or sneezing while packaging the purée	78.6	21.4	92.9	7.1

### 3.3 Pre- and post-training food hygiene practices responses by orange-fleshed sweetpotato purée handlers

Table 3 shows food safety practices by OFSP purée handlers before and after training. Poor handwashing hygiene practices were demonstrated by OFSP purée handlers during the handling of raw OFSP and OFSP purée. Only 64% of all purée handlers washed their hands before handling raw sweetpotato roots and 57% admitted not washing their hands before the puréeing process. Poor handwashing practice by OFSP purée handlers is a source of contamination in OFSP purée processing (Malavi et al., 2018). A significant (*p* < 0.05) increase in frequencies of appropriate food handling practices by OFSP purée handlers was recorded in a post-training hygiene practice assessment.

**Table 3:** Food safety practices of OFSP purée handlers before and after training on food safety.

Food Safety Practices Questions	Before Training		After Training	
	Correct Answers (%)	Wrong Answers (%)	Correct Answers (%)	Wrong Answers (%)
1) Do you wear jewelry while in the sweetpotato purée processing area?	71.4	28.6	85.7	14.3
2) Do you wear nail polish when handling sweetpotato purée?	92.9	7.1	100.0	0.0
3) Do you touch your skin, nose, and other body parts while handling sweetpotato roots and purée?	71.4	28.6	92.9	7.1
4) Do you smoke and chew while working in the purée processing plant?	78.6	21.4	92.9	7.1
5) Do you use detergent whenever you wash your hands?	85.7	14.3	92.9	7.1
6) Do you wash your hands with soap after visiting the toilet?	78.6	21.4	92.9	7.1
7) Do you wear clean apron, hairnet, and safety shoes while in a purée processing plant?	78.6	21.4	100.0	0.0
8) Do you wash your hands before touching and peeling raw sweetpotato roots?	64.3	35.7	92.9	7.1
9) Do you wash your hands before touching cooked sweetpotato roots?	85.7	14.3	100.0	0.0
10) Do you wash your hands before mashing and packaging the purée?	57.1	42.9	92.9	100.0
11) Do you wipe your hands with your apron?	85.7	14.3	92.9	7.1
12) Do you clean your working area before processing sweetpotato purée?	71.4	28.6	100.0	0.0
13) Do you clean freezers used to store processed and packaged sweetpotato purée?	78.6	21.4	100.0	0.0

### 3.4 Effect of training on food safety knowledge and practices of orange-fleshed sweetpotato purée handlers

Table 4 shows pre- and post-training food safety knowledge and practice scores of OFSP purée handlers. Food safety knowledge scores significantly (z = −3.151; *p* = 0.002) increased after training. Food safety practice scores also increased significantly (z = −2.392; *p* = 0.017) after training. There was a significant (*p* < 0.05) improvement in knowledge scores on topics that covered cross-contamination (z = -2.259; *p* = 0.024), cleaning, and sanitation (z = −2.716; *p* = 0.007) after food safety training. Training food handlers on food safety can be an important strategy for enhancing food safety and quality assurance in food industries. A close association has been established between the knowledge and behavior of food handlers (Adesokan et al., 2015). The findings from the current study indicate that training improves food safety knowledge and practices of food handlers. OFSP purée handlers had low levels of knowledge and practices which significantly improved after training. Other research studies have also reported low levels of food safety knowledge and practices by food-handling personnel (Malavi et al., 2017; Soon & Baines, 2012; Park et al., 2010; Roberts et al., 2008). Low level of knowledge was displayed on aspects of cleaning, sanitation, and cross-contamination but significantly (*p < 0.05*) improved after the training. Cleaning and sanitation of equipment are important in preventing cross-contamination (Schlegelova et al., 2010). Before training, more than half of purée handlers did not understand the appropriate times, procedures, and the importance of cleaning and sanitation. This is a risk factor that contributed to purée contamination as reported from the baseline study (Malavi et al., 2018).

**Table 4:** Comparison of pre-and post-training average food safety knowledge and food hygiene practices scores.

Variable	Pre-training scores	Post-training scores	*p-*value
Knowledge of Foodborne Illnesses	85.7 ± 16.2	96.4 ± 9.1	0.057
Knowledge of Personal Hygiene	82.7 ± 17.7	90.8 ± 12.9	0.109
Knowledge of Cleaning and Sanitation	77.4 ± 20.3	96.4 ± 7.1	0.007^a^
Knowledge of Cross-contamination	74.0 ± 18.8	89.0 ± 11.4	0.024^a^
Overall Food Safety Knowledge score	79.6 ± 15.0	91.8 ± 8.2	0.002^a^
Overall Food Hygiene Practices score	76.9 ± 26.6	94.5 ± 9.3	0.017^a^

The values indicate mean ± standard deviation.

a Scores statistically significant at p < 0.05 based on Wilcoxon Signed Ranks Test.

### 3.5 Pre- and post-training hygiene status of equipment surfaces in orange-fleshed sweetpotato purée processing

Table 5 summarizes and compares the mean microbial load from all the equipment surfaces in orange-fleshed sweetpotato purée processing facility before and after the food safety training. Microbial counts on equipment for all parameters tested significantly (*p* < 0.05) reduced to levels acceptable for food processing after the training. Microbial counts from the equipment surfaces in OFSP purée processing after food safety training are shown in Table 6. Aerobic counts (TVC) were not detected from packaging bags but the highest counts with a mean of 4.9 ± 0.1 LOG CFU/cm^2^ were detected from freezers. Yeasts and molds were not detected in 22% of all equipment surfaces sampled but the highest counts were recorded from freezers for storing packaged purée. *S. aureus* was not detected in more than half (56%) of the sampled equipment surfaces but the highest counts (2.7 ± 0.0 LOG CFU/cm^2^) were detected from knives. Enterobacteriaceae and total coliforms were not detected from packaging bags but the highest counts with means of 3.8 ± 0.3 LOG CFU/cm^2^ and 3.6 ± 0.2 LOG CFU/cm^2^ respectively were detected from freezers. In comparison to our baseline study, *E. coli* was not detected on any equipment used in OFSP purée processing after food safety training.

**Table 5:** Pre- and post-food safety training average microbial contamination load on equipment surfaces used in OFSP purée processing (LOG CFU/cm^2^)^a^..

Parameter	Baseline counts (LOG CFU/cm^2^)	Post-training counts (LOG CFU/cm^2^)	*p*-value
Total Viable Counts	7.8 ± 0.4	3.1 ± 0.1	0.00*
Yeast and Molds	5.5 ± 0.2	1.9 ± 0.5	0.00*
*S. aureus*	4.9 ± 0.1	0.7 ± 0.2	0.00*
Enterobacteriaceae	6.6 ± 0.5	2.1 ± 0.1	0.00*
Total coliforms	6.1 ± 0.4	1.9 ± 0.4	0.00*
*E. coli*	3.1 ± 0.2	nd^b^	0.00*

*Mean difference significant at p < 0.05.

a All values represent average microbial counts and standard deviations from food processing equipment surfaces.

b nd-microbial parameter not detected.

**Table 6:** Post-food safety training microbial counts on equipment surfaces in OFSP purée processing plant (LOG CFU/cm^2^)^a^

Sample	TVC	Yeast & Molds	*S. aureus*	Enterobacteriaceae.	Coliforms	*E. coli*
Knives	3.8 ± 0.1 fg	2.1 ± 0.8bc	2.7 ± 0.0b	2.8 ± 0.0cdef	2.7 ± 0.2de	nd*
Tables	3.7 ± 0.2 fg	2.2 ± 0.2bc	1.5 ± 0.3 ab	2.9 ± 0.40def	2.5 ± 0.3cde	nd*
Cooling trays	2.5 ± 0.3de	2.3 ± 0.1bc	nd*	1.9 ± 0.1bcd	1.8 ± 0.1bcd	nd*
Purée machine	3.0 ± 0.5e	2.1 ± 0.0bc	nd*	2.0 ± 0.1bcd	1.6 ± 0.3bcd	nd*
Weighing spoons	3.6 ± 0.0f	nd*	nd*	2.4 ± 0.1defg	2.9 ± 0.0de	nd*
Packaging bags	1.1 ± 0.1b	nd*	nd*	nd*	nd*	nd*
Freezers	4.9 ± 0.1i	3.7 ± 0.1e	1.3 ± 0.2 ab	3.8 ± 0.4 fg	3.6 ± 0.3a	nd*
Cold boxes	1.6 ± 0.4bc	1.6 ± 0.1bc	0.6 ± 0.1 ab	0.9 ± 0.0 ab	0.9 ± 0.0 ab	nd*
Truck	3.8 ± 0.0 fg	3.1 ± 0.0cde	nd*	1.8 ± 0.1bcd	1.1 ± 0.2abc	nd*

*nd-not detected.

a All values represent mean microbial counts and standard deviations. Values with different superscript letters in each column are significantly different (p < 0.05)

Reduction in microbial counts observed after food safety training is attributed to improved food handling practices and implementation of effective cleaning and sanitation procedures as demonstrated and recommended during the food safety training sessions. It is well established that effective cleaning and sanitation reduces up to 99.9% of both spoilage and pathogenic microorganisms from the food processing equipment surfaces (Schlegelova et al., 2010). Appropriate cleaning and sanitation is an important practice for controlling cross-contamination between food and food preparation surfaces. Total counts on most equipment surfaces in this study were below 10^4^ CFU/cm (Ajao & Atere, 2009), a critical value for the formation of biofilms (Ali et al., 2010). Levels below 10^5^ CFU/cm^2^ on equipment have previously been reported in a study that assessed the level of microbial contamination along the frankfurter sausage processing line by Gungor and Gokoglu (2010). The findings from the current study identified that sanitation could not eliminate all microbial species from equipment surfaces. It is interesting to note that even after the training, contamination could occur during the storage of the purée and at dispatch due to relatively high counts of the yeasts and molds and hygiene indicator groups of microorganisms (Enterobacteriaceae family and total coliforms) from the freezers. This is an indication of difficulties in cleaning and sanitation of some internal surface parts of freezers, for instance at the corners, as well as the transfer of contamination from the surface to the internal surface of the freezers during storage and dispatch of the processed purée. Barros et al. (2007) and Ali et al. (2010) assert that several species of yeasts and molds, Enterobacteriaceae and *S. aureus* can adhere and survive on damp equipment surfaces presenting a problem in cleaning and sanitation. Therefore, effective cleaning following documented procedures and the use of approved detergents; sanitation; and draining of equipment to allow drying should be a regular practice in OFSP purée processing to prevent the formation of biofilms and cross-contamination.

### 3.6 Pre- and post-training level of microbial contamination on walls, floors, and drains in orange-fleshed sweetpotato purée processing

Table 7 shows the pre- and post-food safety training mean microbial counts on installations in orange-fleshed sweetpotato purée processing. Microbial counts for most of the parameters from the current study significantly (*p* < 0.05) reduced after food safety training except for *E. coli*. Post-training state of hygiene for walls, floors, and drains in the OFSP purée processing unit is as indicated in Table 8. Low TVC counts were recorded from the floors (4.3 ± 0.2 LOG CFU/cm^2^) while significantly (*p* < 0.05) higher counts (6.2 ± 0.0 LOG CFU/cm^2^) were detected from the drains. All the surfaces were contaminated with yeasts and molds with significantly (*p* < 0.05) lower counts being recorded from walls (1.2 ± 0.1 LOG CFU/cm^2^) and highest counts from drains (4.1 ± 0.0 LOG CFU/cm^2^). *S. aureus* was only detected from walls (1.1 ± 0.1 LOG CFU/cm^2^). This is probably attributed to food handlers occasionally touching the wall surfaces. The lowest Enterobacteriaceae and total coliform counts were detected from the walls with mean counts of 1.8 ± 0.1 and 1.7 ± 0.1 LOG CFU/cm^2^ respectively while high counts were detected from drains with counts of 4.6 ± 0.0 and 3.7 ± 0.0 LOG CFU/cm^2^ respectively. *E. coli* was only detected from drains with mean counts of 2.5 ± 0.0 LOG CFU/cm (Ajao & Atere, 2009).

**Table 7:** Pre- and post-food safety training mean microbial contamination load on installations in orange-fleshed sweetpotato purée processing facility (LOG CFU/cm^2^)^a^ .

Parameter	Baseline counts	Post-training counts	*p*-value
Total Viable Counts	8.5 ± 0.4	5.0 ± 1.0	0.01*
Yeast and Molds	5.8 ± 0.3	3.0 ± 1.6	0.04*
*S. aureus*	5.4 ± 0.1	0.3 ± 0.1	0.00*
Enterobacteriaceae	7.1 ± 0.1	3.3 ± 1.4	0.00*
Total coliforms	6.6 ± 0.2	2.5 ± 1.0	0.00*
*E. coli*	2.6 ± 1.0	0.8 ± 0.2	0.17

*Mean difference significant at p < 0.05.

a All values represent average microbial counts and standard deviations on walls, floors, and drains surfaces in sweetpotato purée processing facility.

**Table 8:** Post-food safety training microbial counts on walls, floors and drains in OFSP purée processing plant (LOG CFU/cm^2^).^a^

Sample	TVC	Yeast & molds	*S. aureus*	Enterobacteriaceae	Coliforms	*E. coli*
Floors	4.3 ± 0.2^ghi^	3.6 ± 0.3^de^	nd*	3.6 ± 0.5^efg^	2.2 ± 0.3^bcde^	nd*
Walls	4.5 ± 0.1^hi^	1.2 ± 0.1^ab^	1.1 ± 0.1^ab^	1.8 ± 0.1^bc^	1.7 ± 0.1^bcd^	nd*
Drains	6.2 ± 0.0^j^	4.1 ± 0.0^e^	nd*	4.6 ± 0.0^g^	3.7 ± 0.0^e^	2.5 ± 0.0^b^

nd*-not detected.

a All values represent mean counts and standard deviations. Values with different superscript letters in each column are significantly different (p < 0.05).

Even though there was a significant reduction in microbial counts from these surfaces, the current level of total counts (10^5^ CFU/cm^2^) after food safety training indicated a need for further improvement in efficiency during the cleaning of walls, floors, and drains. Floors and walls in the purée processing facility were cleaner than the drains due to the adoption of the ‘clean as you go’ principle as recommended during food safety training and regular cleaning of these surfaces before, during, and after production. The drain's surfaces/filters were not large enough for percolating large volumes of wastewater. Frequent water stagnation at these surfaces provided a conducive environment for microbial proliferation thus resulting in higher total counts. Effectual cleaning of floors, walls, and drains is important in controlling the growth and circulation of microbial contaminants in a food processing environment (Ali et al., 2010; Barros et al., 2007). In comparison with the baseline study findings (Malavi et al., 2018), food safety training significantly (*p* < 0.05) improved environmental hygiene in OFSP purée processing.

### 3.7 Pre- and post-training hand hygiene and process water quality in orange-fleshed sweetpotato purée processing

Baseline study findings indicated high microbial contamination levels on personnel hands and in water for processing, hence major sources of contamination in OFSP purée processing (Malavi et al., 2018). Before food safety training, lack of water disinfection program at the facility was reported to have resulted in poor water quality while poor handwashing hygiene led to high microbial counts on personnel's hands (Malavi et al., 2018; Malavi, 2017). Enterobacteriaceae, total coliforms, and *E. coli* counts in water were 6.0, 5.4, and 4.4 LOG CFU/mL respectively before food safety training. Similarly, total counts, yeasts and molds, *S. aureus,* Enterobacteriaceae, total coliforms, and *E. coli* counts from personnel hands were 8.3, 6.0, 5.1, 6.9, 6.6, and 2.7 LOG CFU/cm^2^ respectively. Food safety training on hand hygiene and commendation for water treatment significantly (*p* < 0.05) improved water quality for OFSP purée processing and hand hygiene practices.

Low counts from personnel hands and water were attributed to good handwashing hygiene and regular water treatment (chlorination) as part of the recommendation from the food safety training session. Total counts in treated water were within the acceptable levels recommended by EPA (2012) for use in processing. Yeasts, molds, Enterobacteriaceae, coliforms, and *E. coli* were absent in treated water for handwashing and processing at the facility. A study by Rompre et al. (2002) has reported the chlorination process as being effective in destroying all water quality indicator microorganisms. Contaminated water is a major source of contamination for personnel hands, equipment, and food during processing (Malavi et al., 2018; Liguori et al., 2010).

Low counts from personnel hands indicated improved hand-hygiene practices by food handlers following food safety training. Cross-contamination of food from personnel hands is a significant factor contributing to foodborne disease outbreaks and food spoilage (Green et al., 2006). Training and monitoring of food handler's handwashing hygiene together with the provision of adequate handwashing resources are prime factors for enhancing hand hygiene (Pfuntner, 2011). Hand washing did not, however, eliminate all microorganisms from personnel hands (Montville et al., 2002). Studies thereby recommend the use of disposable gloves and cleaning hands before and after wearing gloves to prevent contamination from personnel hands to foods (Michaels et al., 2004; Montville et al., 2001).

### 3.8 Pre- and post-food safety training microbial quality and safety of orange-fleshed sweetpotato purée

TVC, *S. aureus*, Enterobacteriaceae, and total coliforms in raw OFSP roots were destroyed on steaming. It is well known that adequate heat treatment is effective in reducing microbial load in sweetpotato purée during processing (Perez-Diaz et al., 2008). Yeasts, molds, and *E. coli* (EC) were not detected in raw OFSP roots. *E. coli* was not detected at any of the processing stages but yeasts and molds were detected in the purée after packaging. TVC and *S. aureus* counts increased after cooling, slicing, puréeing, and packaging processes but the increase was only significant (*p* < 0.05) for TVC. Enterobacteriaceae and coliforms were detected in the purée after packaging. Post-processing contamination from OFSP purée handlers and equipment such as knives, cooling trays, puréeing machines, and weighing spoons increased microbial counts in the packaged purée. The counts in the purée were, however, within the limits for use as a food ingredient. Total counts below 10^5^ CFU/g in the purée indicated good keeping quality. This is contrary to a previous study that recorded high microbial counts in OFSP purée (>10^5^ CFU/g) (Malavi et al., 2018). Food safety training significantly (p < 0.05) improved microbial safety and quality of OFSP purée. This was attributed to improved food safety knowledge and handling practices by OFSP purée handlers; improved handwashing hygiene; improved environmental hygiene; enhanced efficiency in cleaning and sanitation; improved water quality; and provision of necessary food safety resources such as handwashing soap and paper towels for hand hygiene after food safety training.

## 4 Conclusion and recommendations

The current study reports findings on the effectiveness of food safety training in food processing in Kenya for the first time. Training in food safety significantly improved food safety knowledge and practices of orange-fleshed sweetpotato purée handlers. Improvement in food safety knowledge and handling practices led to a significant reduction in microbial counts in the purée processing environment hence improved microbial quality of orange-fleshed sweetpotato purée. However, frequent food safety education, monitoring, and management support through the provision of necessary food safety resources are key factors for enhancing quality and safety in food processing of orange-fleshed sweetpotato purée.

## CRediT authorship contribution statement

**Derick Nyabera Malavi:** Formal analysis, Data curation, Conceptualization, Methodology, Writing - original draft, Writing - review & editing. **George Ooko Abong’:** Conceptualization, Supervision, Writing - review & editing. **Tawanda Muzhingi:** Conceptualization, Supervision, Writing - review & editing.

## Declaration of competing interest

The authors declare no conflict of interest.
